# Well-Defined Dual Light- and Thermo-Responsive Rod-Coil Block Copolymers Containing an Azobenzene, MEO_2_MA and OEGMA

**DOI:** 10.3390/polym12020284

**Published:** 2020-02-01

**Authors:** Changjun Park, Jaewon Heo, Jinhee Lee, Taehyoung Kim, Sang Youl Kim

**Affiliations:** Department of Chemistry, Korea Advanced Institute of Science and Technology (KAIST), Daejeon 34141, Korea; pcjhenry@kaist.ac.kr (C.P.); pegasus64@kaist.ac.kr (J.H.); solwal@kaist.ac.kr (J.L.); thkim93@kaist.ac.kr (T.K.)

**Keywords:** light-responsive behavior, thermo-responsive behavior, rod-coil block copolymer, controlled polymerization, self-assembly, azobenzene, MEO_2_MA, OEGMA

## Abstract

Here we report the dual light- and thermo-responsive behavior of well-defined rod-coil block copolymers composed of an azobenzene unit, 2-(2-methoxyethoxy)ethyl methacrylate (MEO_2_MA) and oligo(ethylene glycol) methacrylate (OEGMA). Azobenzene-containing rigid rod blocks prepared by chain growth condensation polymerization of the azobenzene containing monomer were used as a macroinitiator of atom transfer radical polymerization (ATRP) after attaching an α-bromoisobutyryl group as an end group. Synthesis of well-defined rod-coil block copolymers with different coil block lengths was achieved by copolymerization of MEO_2_MA and OEGMA monomers. The synthesized polymers exhibited amphiphilic properties and polymeric micelles were formed in aqueous solution. The light-responsive behaviors of azobenzene moieties, photoisomerization by irradiation of light, and thermo-responsive behaviors of P(MEO_2_MA-*co*-OEGMA) coil blocks, aggregation by increment of temperature over lower critical solution temperature, were investigated. A dual stimuli-responsive behavior of the rod-coil block copolymers was observed when exposed to light and heat.

## 1. Introduction

Self-assembly of block copolymers consisting of two immiscible polymer chains has received considerable attentions owing to their ability to form well-defined structures composed of polymeric materials [1,2]. These block copolymers self-assemble with minimization of the thermodynamic energy of two immiscible blocks with kinetic manipulation. Supramolecular assembly of polymeric materials can provide numerous structures with various morphologies including micelles, vesicles and fibers [3,4]. In particular, self-assembly behavior of rod-coil block copolymers attracts significant interest due to the interesting conformational asymmetry between a rigid rod block and a flexible coil block [5,6]. In addition, microphase separation is promoted compared with coil-coil block copolymers owing to the high stiffness difference between rod and coil blocks. To construct complicated systems with precise control, syntheses of well-defined polymers is required through controlled polymerization techniques such as chain-growth condensation polymerization (CGCP) [7,8] and atom transfer radical polymerization (ATRP) [9,10].

Stimuli-responsive polymeric materials are interesting materials that can tune their properties in multiple ways when exposed to external stimuli [11,12]. Among the external stimuli, light has received considerable attention since light irradiation can be focused onto specific areas from outside the system [13,14]. In addition, light-responsive behaviors can be achieved by irradiation of light only, without using additional reagents. Furthermore, the degree of light-responsive behaviors can be controlled precisely by adjusting the light intensity or wavelength. Azobenzene is one of the most intriguing photochromic molecules which undergoes a *trans*-to-*cis* isomerization by irradiation with UV light and reversible *cis*-to-*trans* isomerization occurs by irradiation with visible light or application of heat [15,16]. Due to the remarkable change of dipole moment and molecular configuration during the reversible isomerization, azobenzene derivatives have been extensively studied in various areas, including supramolecular chemistry, materials science and biological science [17,18]. The light-responsive behavior of azobenzene moieties incorporated into polymer chains have been known to induce reversible modulation of their mechanical, morphological and polar properties by irradiation of light [19,20,21]. Another readily available physical stimulus is heat, and thermo-responsive polymers have been extensively investigated because of their relation to biotechnology and nanotechnology [22,23]. In addition, a lower critical solution temperature (LCST) of hydrophilic or amphiphilic polymers in water is especially important for biomedical applications such as drug delivery. Poly(*N*-isopropylacrylamide) (PNIPAM), which exhibit a LCST about 32 °C in water, is one of the well-known thermo-responsive polymers [24,25]. PNIPAM has been intensively studied due to its biocompatibility and maintenance of LCST against environmental changes including pH and concentration. Recently, the copolymerization of two oligo(ethylene glycol) monomers with different chain lengths has been investigated to synthesize thermo-responsive polymers with an adjustable LCST [26,27]. LCST values of random copolymers composed of 2-(2-methoxyethoxy)ethyl methacrylate (MEO_2_MA) and oligo(ethylene glycol) methacrylate (OEGMA) vary from 26 to 90 °C with precise control by modulation of co-monomer composition. Moreover, a uniform thermal profile compared with PNIPAM was reported [28].

Polymers containing the both light-responsive moieties and thermo-responsive moieties are expected to exhibit dual stimuli-responsive behaviors against change of temperature and irradiation of light [29,30]. The first example of a light- and thermo-responsive polymer was achieved based on *N*-(4-phenylazophenyl)acrylamide and NIPAM [31]. Interestingly, the LCST of the polymer was increased by irradiation of UV light due to the change in the dipole moment based on *trans*-to-*cis* isomerization of the azobenzene units. So far, numerous studies about dual light- and thermo-responsive polymers have been reported [32,33,34,35,36,37,38]. However, most of the research on light- and thermo-responsive block copolymers is limited to the case of coil-coil block copolymers [39,40,41], and the investigation of the dual stimuli-responsive behaviors of rod-coil block copolymers is hindered mainly due to the synthetic difficulty in preparing these compounds. Herein, dual stimuli-responsive rod-coil block copolymers (DRBCP) containing azobenzene moieties in main chain of rod block and MEO_2_MA and OEGMA in coil block were designed and synthesized. The combination of the CGCP and ATRP produces rod-coil block copolymers with well-defined structures. After the polymerization, the self-assembly behavior of the prepared polymers in aqueous solution was investigated to demonstrate dual stimuli-responsive behavior of the rod-coil block copolymers.

## 2. Experimental

### 2.1. Materials and Methods

4-Hydroxybenzyl alcohol (99%), α-bromoisobutyryl bromide (98%), triethylamine (TEA) (99.5%), tetrahydrofuran (THF) (99.9%), copper(I) chloride (CuCl) (99.995%), copper (II) chloride (CuCl_2_) (99.999%), 2,2′-bipyridine (bpy) (99%), MEO_2_MA (95%) and OEGMA (*M*_n_ = 475 g/mol) were purchased from Sigma-Aldrich (St. Louis, MO, USA). MEO_2_MA and OEGMA were passed through a column filled with neutral alumina to remove inhibitors. Potassium carbonate (99.5%), *N*,*N*-dimethylformamide (DMF) (99.5%) and anisole (98%) were purchased from Junsei Chemical Co., Ltd. (Tokyo, Japan). Potassium carbonate was dried *in vacuo* at 150 °C for 24 h prior to use. DMF and anisole were stirred in the presence of calcium hydride overnight and subsequently distilled under reduced pressure prior to use. All other chemicals were used as received. Poly(arylene ether azobenzene) (PAEAz) as a rod block of rod-coil block copolymers was synthesized through CGCP according to a literature procedure [42].

The ^1^H nuclear magnetic resonance (NMR) spectra of the synthesized materials were recorded on an Avance 400 MHz NMR spectrometer (Bruker, Billerica, MA, USA). Molecular weights and molecular weight distributions of polymers were measured on a T60A gel permeation chromatography (GPC) system (Viscotek, Malvern, UK) with PLgel 10 µm MIXED-B as column packing. The GPC measurements were conducted using THF as an eluent at a rate of 1 mL/min at 35 °C with calibration relative to linear polystyrene or poly(methyl methacrylate) standards. All samples were filtered through a 0.45 µm syringe filter before the measurements. Thermogravimetric analysis (TGA) was performed on a TGA Q50 system (TA Instruments, New Castle, DE, USA) with a heating rate of 10 °C/min in nitrogen. Differential scanning calorimetry (DSC) was performed on a TA Instruments DSC Q20 with a heating rate of 5 °C/min in nitrogen. For the preparation of micellar aggregates through self-assembly of DRBCPs in aqueous solution, aliquots of the polymer in THF solution were added into water and stirred for 3 h with evaporation of THF. Concentration of the aqueous solution was precisely controlled by the amounts of injected THF. Dynamic light scattering (DLS) measurements were performed on a 90Plus/BI-MAS particle size analyzer (Brookhaven Instruments Corp., Holtsville, NY, USA) at wavelength of 658 nm. The scattering angle used for the measurements was 90°. The CONTIN approximation was used to convert the diffusion coefficient into the hydrodynamic diameter. Field-emission Scanning electron microscopy (FE-SEM) was performed on a Magellan 400 FE-SEM (FEI, Hillsboro, OR, USA). Samples were prepared on a silicon wafer by drop casting and coated with Pt before imaging. Irradiation of light was performed using a SUV-DC-P (Lumatec, Deisenhofen, Germany) as a light source. To irradiate UV light, a filter having transmittance range from 315 nm to 390 nm was placed in front of the light source. To irradiate visible light, a filter having 400 nm of cut-off wavelength was placed in front of the light source. Samples were placed at 3 cm from the lamp and external light was prevented. UV-vis spectroscopy was conducted using a V-530 spectrometer (Jasco Inc., Tokyo, Japan) equipped with a ETC-505T temperature controller.

### 2.2. Synthetic Procedures

#### 2.2.1. End Group Modification of PAEAz with Terminal Hydroxyl Group (PAEAzOH)

A dried three-neck flask was charged with PAEAz (0.49360 g, 0.24 mmol), 4-hydroxybenzyl alcohol (0.05960 g, 0.48 mmol), potassium carbonate (0.06634 g, 0.48 mmol) and DMF (10 mL) with equipment of a mechanical stirrer and a Dean-Stark trap. The reaction mixture was heated at 70 °C for 2 h under nitrogen atmosphere. After reaction, the reaction mixture was poured into distilled water and slightly acidified. The crude product was filtered and PAEAzOH as a yellow solid (0.512 g, 97.0%) was obtained through reprecipitation in methanol. *M*_n_: 2.20 × 10^3^ g/mol. PDI: 1.126. ^1^H-NMR (THF-*d*_8_, 400 MHz, ppm): 8.36 (s, 6H), 8.26 (m, 2H), 8.19 (dd, 6H), 8.08 (m, 14H), 8.02 (d, 1H), 7.62 (d, 1H), 7.44 (d, 2H), 7.43 (d, 1H), 7.35 (dm, 20H), 7.12 (d, 2H), 7.10 (t, 1H), 4.61 (d, 2H), 4.25 (t, 1H).

#### 2.2.2. Synthesis of Macroinitiator with Terminal Bromide Group (PAEAzOBr)

PAEAzOH (0.49940 g, 0.227 mmol) and TEA (0.04594 g, 0.454 mmol) were dissolved in 10 mL of THF. α-Bromoisobutyryl bromide (0.10438 g, 0.454 mmol) was slowly added into the solution by gastight syringe at 0 °C. The reaction mixture was stirred at room temperature for 24 h. After reaction, the reaction mixture was poured into distilled water. The crude product was filtered, washed with distilled water and methanol, and dried *in vacuo*. PAEAzOBr as an orange solid (0.487 g, 91.3%) was obtained through reprecipitation in hexane. *M*_n_: 2.35 × 10^3^ g/mol. PDI: 1.112. ^1^H-NMR (THF-*d*_8_, 400 MHz, ppm): 8.36 (s, 6H), 8.26 (m, 2H), 8.19 (dd, 6H), 8.08 (m, 14H), 8.02 (d, 1H), 7.62 (d, 1H), 7.44 (d, 2H), 7.43 (d, 1H), 7.35 (dm, 20H), 7.12 (d, 2H), 7.10 (t, 1H), 5.23 (s, 2H), 1.94 (s, 6H).

#### 2.2.3. General Polymerization Procedure of PAEAz-*b*-P(MEO_2_MA-*co*-OEGMA) (DRBCP)

A dried Schlenk flask was charged with PAEAzOBr (0.1024 g, 0.04 mmol), CuCl (9.5 mg, 0.096 mmol), CuCl_2_ (2.16 mg, 0.016 mmol), bpy (78.46 mg, 0.192 mmol), MEO_2_MA (2.0316 g, 10.8 mmol), OEGMA (0.540 g, 1.2 mmol) and anisole (2.75 mL) under nitrogen atmosphere. The reaction mixture was degassed by three “freeze-pump-thaw” cycles and heated at 90 °C or 100 °C. After reaction, the reaction mixture was cooled and diluted with THF. The solution was passed through a column filled with neutral aluminum oxide and evaporated. The crude product was purified by dialysis in distilled water for a week and DRBCP as an orange sticky solid was obtained after evaporation and drying *in vacuo*. ^1^H-NMR (THF-*d*_8_, 400 MHz, ppm): 8.31, 8.08, 8.01, 7.29, 7.24, 7.20, 7.17, 6.96, 5.00, 4.10, 3.67, 3.63, 3.54, 3.38, 1.90, 1.80 1.23, 1.03, 0.86.

## 3. Results and Discussion

### 3.1. Syntheses of DRBCPs

**Scheme 1:**
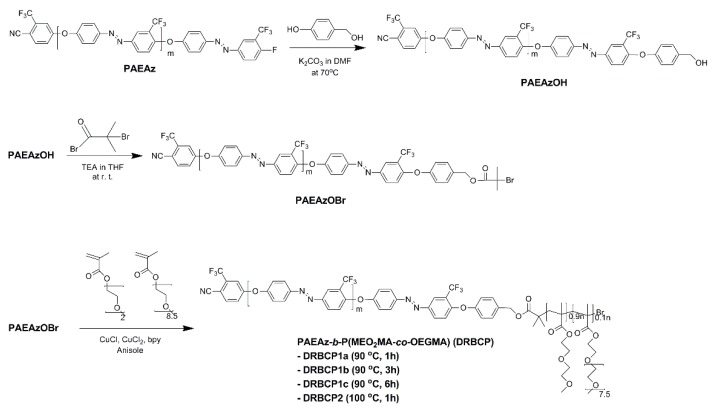
Syntheses of DRBCPs from PAEAz rod blocks through end group modification and subsequent ATRP. DRBCPs with different length of the coil blocks were synthesized by varying the reaction temperature and time of ATRP.

The synthetic routes of DRBCPs are shown in Scheme 1. The PAEAz rod block with a narrow molecular weight distribution was synthesized by CGCP according to the reported procedure [42]. End group modification of PAEAz through reaction with 4-hydroxybenzyl alcohol and subsequent reaction with α-bromoisobutyryl bromide were carried out to introduce initiating sites for ATRP. Feed ratio of MEO_2_MA and OEGMA for ATRP was set to 9:1 for preparation of the polymers exhibiting LCST about 37 °C [26]. Herein, four DRBCPs with different length of P(MEO_2_MA-*co*-OEGMA) coil blocks were prepared through ATRP. These polymers are classified according to their reaction temperature and time. First, DRBCPs are assorted by respective reaction temperature. DRBCPs synthesized at 90 °C are labelled as DRBCP1s, and DRBCP synthesized at 100 °C is labelled as DRBCP2. In the case of DRBCP2, the polymerization was carried out for 1 h. Subsequent classification of DRBCP1s is based on the reaction time. The synthesized polymers with the reaction time for 1 h, 3 h and 6 h are named as DRBCP1a, DRBCP1b and DRBCP1c, respectively.

**Figure 1:**
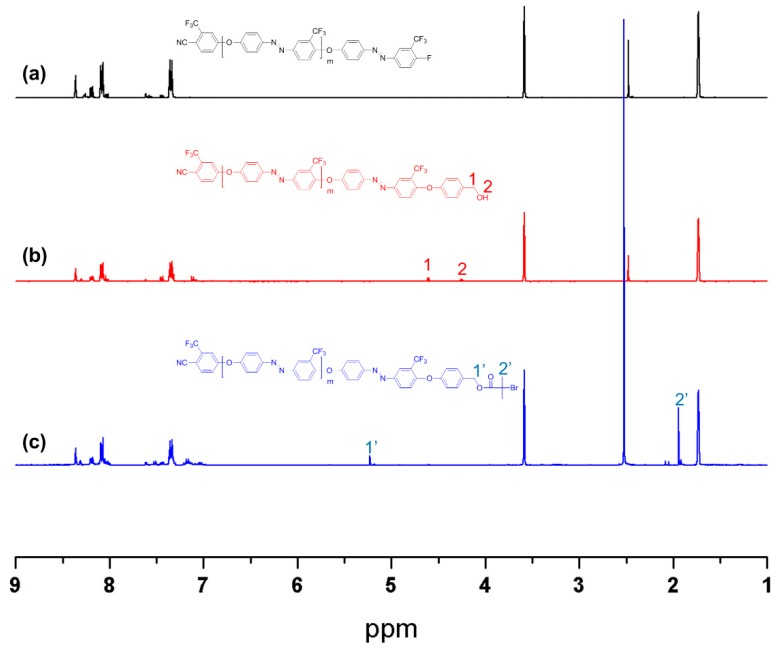
^1^H NMR spectra of (**a**) PAEAz. (**b**) PAEAzOH. (**c**) PAEAzOBr in THF-*d*_8_.

^1^H-NMR spectra of PAEAz rod blocks with different end groups were obtained to confirm the incorporation of functional groups (Figure 1). Before end group modification, the corresponding peaks of the aromatic protons from PAEAz were observed. After the end group modification to introduce hydroxyl groups, the peaks at 4.61 ppm and 4.25 ppm referring to benzyl alcohol end groups appeared. In the case of PAEAzOBr macroinitiator, the peak of benzyl alcohol end groups at 4.61 ppm shifted to 5.23 ppm and the peak at 4.25 ppm corresponding to the hydroxyl protons disappeared. On the other hand, a new peak at 1.94 ppm ascribed to the methyl protons of α-bromoisobutyryl bromide incorporated to PAEAz appeared, indicating the successful synthesis of macroinitiator for ATRP. In addition, polydispersity of the polymers was measured by GPC and the results reveal that the macroinitiator with a narrow molecular weight distribution was synthesized (Figure S1). Molecular weights and polydispersity of the PAEAz derivatives are listed in Table 1.

**Figure 2:**
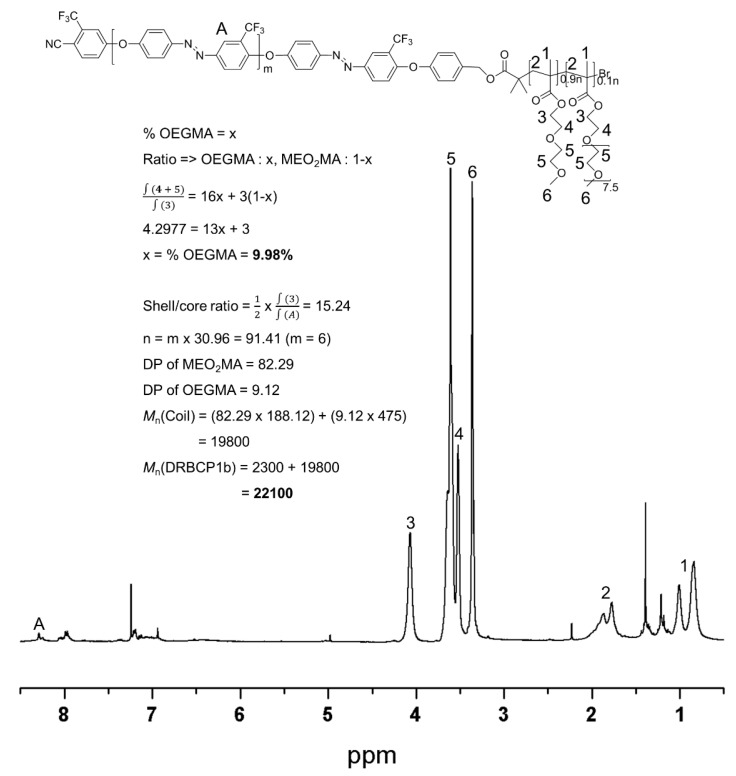
^1^H-NMR spectrum with calculation of % OEGMA and molecular weight of DRBCP1b in chloroform-*d*.

As mentioned above, P(MEO_2_MA-*co*-OEGMA) coil blocks were synthesized by ATRP of MEO_2_MA and OEGMA with the PAEAzOBr macroinitiators to produce dual light- and thermo-responsive PAEAz-*b*-P(MEO_2_MA-*co*-OEGMA)s, named as DRBCPs. The polymerization was carried out by using CuCl, CuCl_2_ and bpy in anisole. ^1^H-NMR spectra of DRBCPs were obtained using chloroform-*d* (CDCl_3_) (Figure 2 and Figures S2–S4) to measure correct molecular weight of the synthesized polymers without overlapping of solvent residual peaks and the peaks of coil block, occurred in THF-*d*_8_. DRBCPs were dissolved well in CDCl_3_ and the peaks corresponding to the aromatic protons of PAEAz at 8.31 ppm and the aliphatic protons of the coil block from 0.5 ppm to 4.5 ppm were clearly observed. The incorporation ratio of MEO_2_MA and OEGMA was calculated prior to the estimation of the molecular weights of DRBCPs. The percentage of OEGMA (% OEGMA) was determined from the integration of protons adjacent to the ethereal oxygens except the end methyl protons (4 + 5) and the protons conterminous to the oxygen of ester group (3). The molecular weights of DRBCPs were obtained by end-group analysis from the integration of the aromatic protons for PAEAz (A) and the protons adjacent to the oxygen of ester group (3) by using the estimated incorporation ratio of MEO_2_MA and OEGMA. In the case of DRBCP1s, the molecular weights of the coil blocks were proportionally increased with increment of reaction time, indicating that the polymerization of DRBCPs proceeded in a controlled manner.

Molecular weight distributions of DRBCPs were also measured by GPC (Figure S5). Molecular weights measured by GPC were relatively low compared to the molecular weights measured by ^1^H- NMR and the difference between *M*_n_ (NMR) and *M*_n_ (GPC) gradually increased with increase of coil block ratio in DRBCPs due to the interaction of polar coil blocks, especially OEGMA, with the columns. 

Although the GPC analysis did not predict molecular weights precisely, narrow polydispersities were obtained, indicating that well-defined DRBCPs were prepared by controlled polymerizations. Measurement of the thermal properties were performed by TGA and DSC. The 5% weight loss temperatures (*T*_d5_) were obtained in the range of 234–309 °C with tendency to decrease by increment of the length of coil blocks (Figure S6). All of the DRBCPs exhibited the glass transition temperatures (*T*_g_) around −35 °C (Figure S7). Details on the characterization of the DRBCPs are summarized in Table 2.

### 3.2. Self-Assembly Behaviors in Aqueous Solution

**Figure 3:**
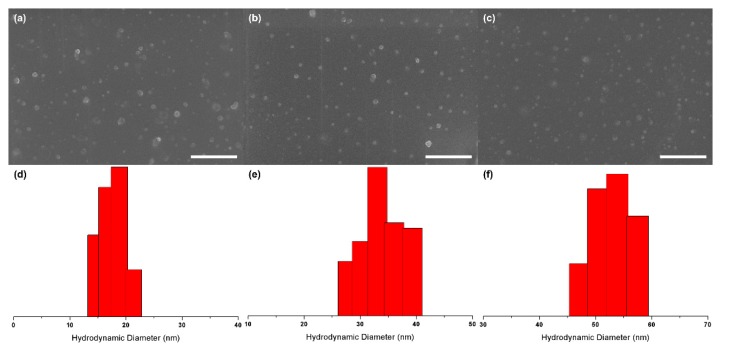
(**a**–**c**) FE-SEM images (scale bar, 500 nm) and (**d**–**f**) DLS plots of the self-assembled structures of DRBCPs in aqueous solution. (**a**,**d**) DRBCP1b. (**b**,**e**) DRBCP1c. (**c**,**f**) DRBCP2.

The synthesized DRBCPs exhibit amphiphilic properties due to the presence of hydrophobic PAEAz rod blocks and hydrophilic P(MEO_2_MA-*co*-OEGMA) coil blocks. Therefore, the self-assembly behaviors of the DRBCPs in aqueous solution were investigated. Supramolecular assemblies of DRBCPs in aqueous solution were induced by addition of THF solution of the DRBCPs into water followed by evaporation to remove THF. In the case of DRBCP1a, the polymer was insoluble in water due to the short length of hydrophilic coil blocks. Consequently, self-assembly behaviors were monitored for DRBCP1b, DRBCP1c and DRBCP2. Morphology of the aggregates composed of DRBCPs was visualized by FE-SEM (Figure 3a–c). FE-SEM images of the aggregates revealed that micellar structures were formed in aqueous solution, indicating that hydrophobic PAEAz rod blocks were aggregated as a core of polymeric micelles. In addition, DLS study of the self-assembled structures of DRBCPs exhibited the generation of the polymeric micelles with quite narrow distributions (Figure 3d–f). The hydrodynamic diameter of micellar aggregates increased from 18.9 nm to 54.0 nm with increase of the molecular weight of DRBCPs.

### 3.3. Light-Responsive Behaviors

**Figure 4:**
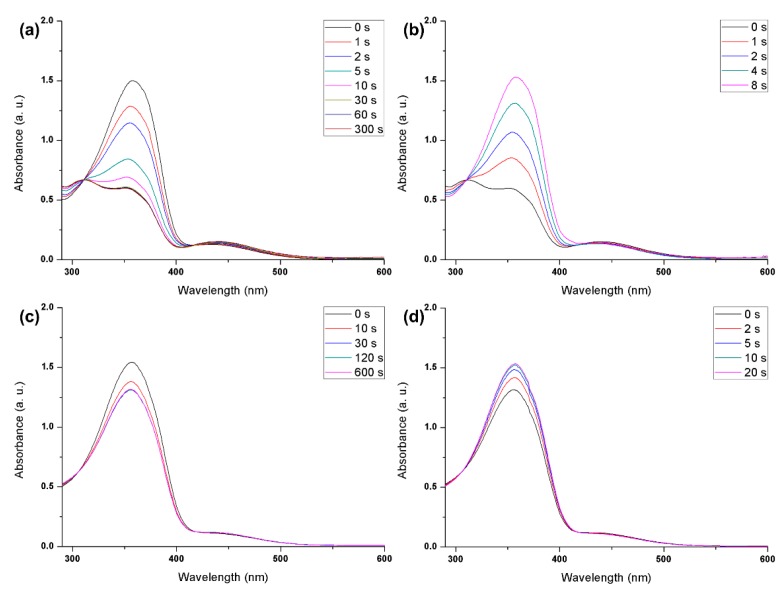
UV-vis absorption spectral changes of DRBCP1b (**a**,**b**) in THF solution and (**c**,**d**) in aqueous solution. Irradiation of (**a**,**c**) UV light and (**b**,**d**) visible light subsequently were conducted.

The light-responsive isomerization behavior of the azobenzene derivatives incorporated into DRBCPs were investigated by UV-vis spectroscopy to monitor absorption spectral changes (Figure 4 and Figures S8 and S9). THF solutions and aqueous solutions of DRBCPs with an appropriate concentration to prevent an excess of absorption intensity over the measurable limit were prepared. Photoisomerization from *trans*-azobenzene to *cis*-azobenzene was achieved by irradiation of UV light with a decrease of the absorption bands at 360 nm corresponding to the π–π* transition of the *trans*-azobenzene and simultaneous increase of the absorption bands at 440 nm referring to the n–π* transition of the *cis*-azobenzene [43] (Figure 4a). In addition, increase of the absorption bands at 360 nm and decrease of the absorption bands at 440 nm was observed by subsequent irradiation of visible light for a few seconds, indicating reversible manner of the photoisomerization of azobenzene moieties (Figure 4b). Compared with the light-responsive behaviors of DRBCPs in THF solution, relatively low degree of reversible photoisomerization was shown in aqueous solution of DRBCPs (Figure 4c,d). This result reveals that azobenzene moieties of DRBCPs in THF solution are unrestrained due to much higher solubility of the PAEAz rod blocks than that in aqueous solution. Light-responsive isomerization of the PAEAz rod blocks incorporated to DRBCPs in aqueous solution was restricted by aggregation of the hydrophobic rod blocks. Therefore, difference of absorption spectra between DRBCPs in THF solution and in aqueous solution is another evidence of construction of the polymeric micelle structures in aqueous solution.

### 3.4. Thermo-Responsive Behaviors

**Figure 5:**
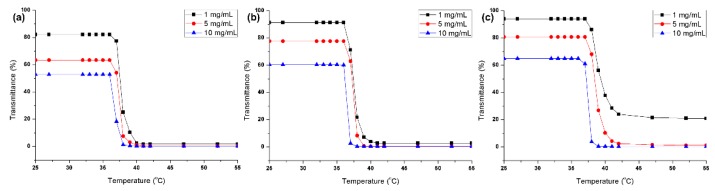
Temperature-dependent transmittance changes of DRBCPs in aqueous solution with varying concentration. (**a**) DRBCP1b. (**b**) DRBCP1c. (**c**) DRBCP2.

The thermo-responsive phase transition behaviors of P(MEO_2_MA-*co*-OEGMA) coil blocks incorporated into DRBCPs were monitored by UV-vis spectroscopy with control of temperature to observe LCST of the aqueous solution. Figure 5 shows the temperature-dependent transmittance changes of DRBCPs in aqueous solution with varying concentration from 1 mg/mL to 10 mg/mL at 600 nm. All of the DRBCP aqueous solutions revealed LCST around 37 °C due to the incorporation ratio of MEO_2_MA and OEGMA controlled by the feed ratio [26]. As shown in Figure 2 and Figures S3 and S4, the DRBCPs with slightly different incorporation ratio of OEGMA in the coil block were synthesized. LCST of the aqueous solutions tended to be raised by increment of the incorporation ratio of OEGMA with higher solubility in water, regardless of the length of coil block [26,28]. In addition, each DRBCP in aqueous solution with higher concentration exhibited lower LCST, which is consistent with literature [44,45]. 

Table 3 shows LCST of DRBCPs with various incorporation ratio of OEGMA in the coil block and concentration. To check reversibility of thermo-responsive behaviors, heating over LCST and subsequent cooling under LCST of the DRBCP aqueous solutions were performed. All of the solutions exhibited the recovery of initial transmittance with uniform thermal profile which originate from the characteristics of P(MEO_2_MA-*co*-OEGMA) [28] (Figure S10). These thermo-responsive behaviors were observed by naked eye due to the significant change of the transmittance (Figure S11).

The correlation between transmittance and concentration of DRBCPs in aqueous solution was investigated to decide appropriate condition of the solutions for further demonstration of dual stimuli-responsive behaviors. As shown in Figure 5, the initial transmittance of the aqueous solution was reduced by increase of the concentration or decrease of the ratio of coil block due to increment of the ratio of hydrophobic PAEAz rod block. In the case of 10 mg/mL solution, each DRBCP solution exhibited relatively low initial transmittance in the range of 50–60%. Consequently, the aqueous solutions with lower concentration were considered to increase transmittance change between temperature under and over LCST. In the case of 1 mg/mL solution, however, the aqueous solution of DRBCP2 exhibited relatively high transmittance over LCST compared with other aqueous solutions of DRBCPs, suggesting that intramicellar aggregation of coil blocks were occurred. Transmittance of DRBCP2 aqueous solution over LCST was inclined to decrease by increment of concentration and the comparable result was obtained with the 5 mg/mL solution. Therefore, the 5 mg/mL solution of each DRBCP was prepared to demonstrate dual stimuli-responsive behaviors.

### 3.5. Dual Stimuli-Responsive Behaviors

**Figure 6:**
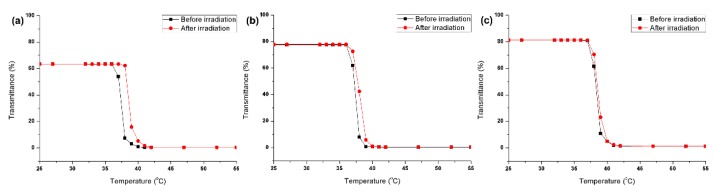
Dual stimuli-responsive behaviors of DRBCP micelles in aqueous solution at 5 mg/mL concentration. (**a**) DRBCP1b. (**b**) DRBCP1c. (**c**) DRBCP2.

As noted above, 5 mg/mL aqueous solutions of DRBCP1b, DRBCP1c, DRBCP2 were used to study dual stimuli-responsive behaviors. Dual stimuli-responsive behaviors were monitored by measurement of difference between LCST before and after irradiation of UV light for 10 min (Figure 6). 

**Scheme 2:**
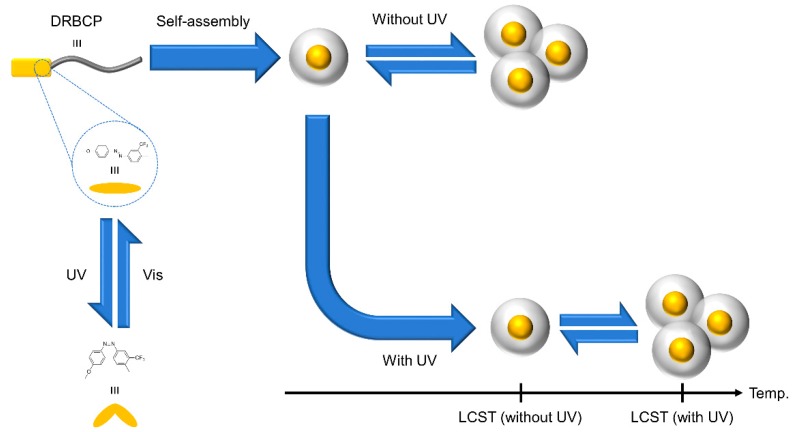
Schematic illustration of the self-assembly and the response to light and temperature of dual stimuli-responsive rod-coil block copolymers (DRBCPs).

All of the aqueous solutions exhibited an increase of LCST by irradiation of UV light, but the degree of change was different. DRBCP2 revealed slight change of LCST, 0.2 °C, and DRBCP1c exhibited 0.7 °C of LCST change. However, DRBCP1b displayed 1.2 °C of LCST change and the result is relatively high compared with other DRBCP solutions. Tendency to rise increment of LCST by irradiation of UV light was found with diminution of the length of coil block. Contraction of the chain length of coil block gave rise to an increase of the ratio of azobenzene moieties in DRBCPs. As a result, the amount of LCST change increased owing to the enhancement of the effect of polarity change arised from conformational change of azobenzene derivatives by irradiation of UV light [21]. Schematic illustration of the self-assembly and the response to light and temperature of dual stimuli-responsive rod-coil block copolymers (DRBCPs) is shown in Scheme 2.

## 4. Conclusions

In summary, we have demonstrated the dual stimuli-responsive behaviors of some well-defined block copolymers, DRBCPs, composed of light-responsive PAEAz rod blocks and thermo-responsive poly(MEO_2_MA-*co*-OEGMA) coil blocks. Narrow molecular weight distributions of DRBCPs were achieved by combination of CGCP and ATRP. DRBCPs formed micellar structures through self-assembly in aqueous solution due to their amphiphilic properties. The length of the hydrophilic coil block should be sufficiently long to form micelles in aqueous solution with enough solubility. The polymers’ light-responsive behavior and thermo-responsive behavior were investigated by irradiation of light and control of temperature nearby LCST, respectively. The relatively low degree of photoisomerization in aqueous solution compared with the degree of photoisomerization in THF solution originated from the restrained movements of rod blocks located at core of the polymeric micelles. Significant decrease of transmittance was observed at temperatures over LCST through intermicellar aggregation of coil blocks and reversible micelle segregation by cooling under LCST with a uniform thermal profile was also observed. The rod-coil block copolymers showed dual stimuli-responsive behavior, with an increase of LCST by irradiation of UV light, owing to polarity change induced by conformational change of azobenzene moieties. Further investigation on morphology change and stimuli-responsive behaviors of corresponding self-assembled structures composed of dual light- and thermo-responsive rod-coil block copolymers is underway.

## Figures and Tables

**Table 1:** Molecular weights and PDI of the PAEAz derivatives.

Polymer	*M*_n_ (NMR) ^1^	*M*_n_ (GPC) ^2^	*M*_w_ (GPC) ^2^	PDI ^2^
PAEAz	2091	3264	3691	1.13
PAEAzOH	2196	3305	3723	1.13
PAEAzOBr	2345	3243	3607	1.11

^1^ Calculated by end-group analysis. ^2^ Determined by THF-GPC using polystyrene standards.

**Table 2:** Characterization of the DRBCPs.

Polymer	*M*_n_ (NMR) ^1^	*M*_n_ (GPC) ^2^	*M*_w_ (GPC) ^2^	PDI ^2^	*T*_d5_ (°C) ^3^	*T*_g_ (°C) ^4^
DRBCP1a	9200	5760	6410	1.11	309	−34.6
DRBCP1b	22,100	12,300	13,900	1.13	299	−35.5
DRBCP1c	42,100	14,600	16,600	1.13	250	−34.2
DRBCP2	60,200	18,400	20,700	1.13	234	−35.2

^1^ Calculated by end-group analysis. ^2^ Determined by THF-GPC using poly(methyl methacrylate) standards. ^3^ Measured by TGA with a heating rate of 10 °C/min under nitrogen flow. ^4^ Measured by DSC at the second scan with a heating rate of 5 °C/min under nitrogen flow.

**Table 3:** LCST of DRBCPs with various incorporation ratio of OEGMA and concentration.

Polymer	OEGMA Ratio (%) ^1^	LCST (°C) ^2^		
		1 mg/mL	5 mg/mL	10 mg/mL
DRBCP1b	9.98	37.7	37.5	36.8
DRBCP1c	9.18	37.5	37.4	36.5
DRBCP2	10.90	38.9	38.7	37.5

^1^ Calculated by ^1^H-NMR spectra with end-group analysis. ^2^ Measured by UV-vis spectroscopy with control of temperature.

## Supplementary Materials

The following are available online at https://www.mdpi.com/2073-4360/12/2/284/s1, Figure S1: THF-GPC profiles of PAEAz derivatives, Figures S2–S4: ^1^H NMR spectra with calculation of % OEGMA and molecular weights of DRBCPs, Figure S5: THF-GPC profiles of DRBCPs, Figure S6: TGA curves of the DRBCPs, Figure S7: DSC diagrams of the DRBCPs, Figures S8 and S9: UV-vis absorption spectral changes of DRBCPs, Figure S10: Reversible thermo-responsive behaviors of DRBCPs in aqueous solution. Figure S11: Visualization of transmittance change by controlling temperature.
