# Supplementary material for: Well-Defined Dual Light- and Thermo-Responsive Rod-Coil Block Copolymers Containing an Azobenzene, MEO2MA and OEGMA
Source: Polymers (Basel). 2020 Feb 1;12(2):284. doi: 10.3390/polym12020284 (PMC7077463; doi:10.3390/polym12020284)
Supplement: Supplementary file 1 [file polymers-12-00284-s001.pdf]

Supplementary Materials

Well-Defined Dual Light- and Thermo-Responsive Rod-Coil Block Copolymers Containing an Azobenzene, MEO_2MA and OEGMA

Changjun Park [1], Jaewon Heo [1], Jinhee Lee [1], Taehyoung Kim [1] and Sang Youl Kim [1,*]

[1] Department of Chemistry, Korea Advanced Institute of Science and Technology (KAIST), Daejeon 34141, Korea.

* Correspondence: kimsy@kaist.ac.kr; Tel.: (+)82-42-350-2834.

Supplementary Figures

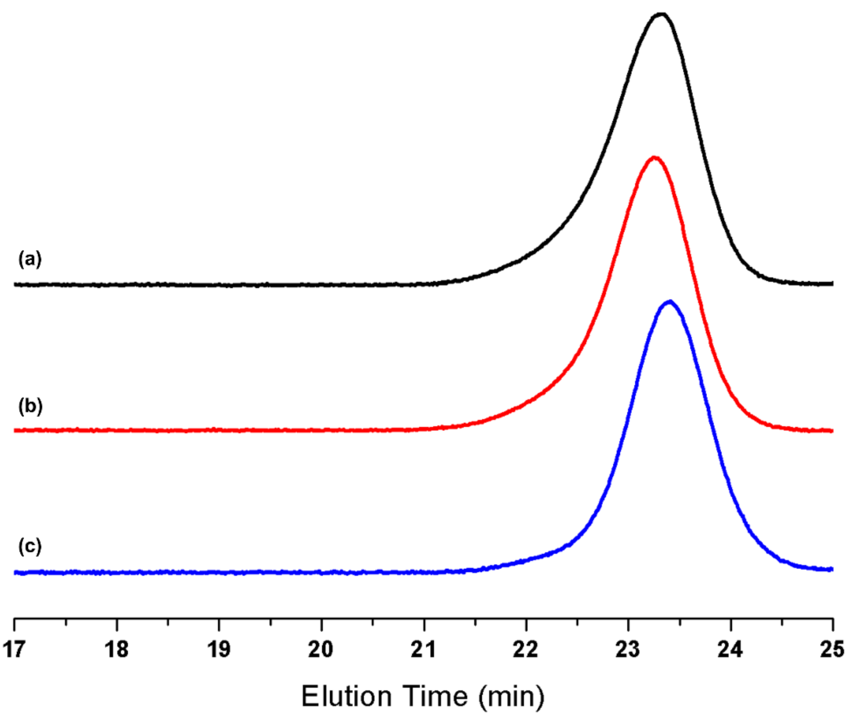

Figure S1. THF-GPC profiles of (a) PAEAz. (b) PAEAzOH. (c) PAEAzOBr.

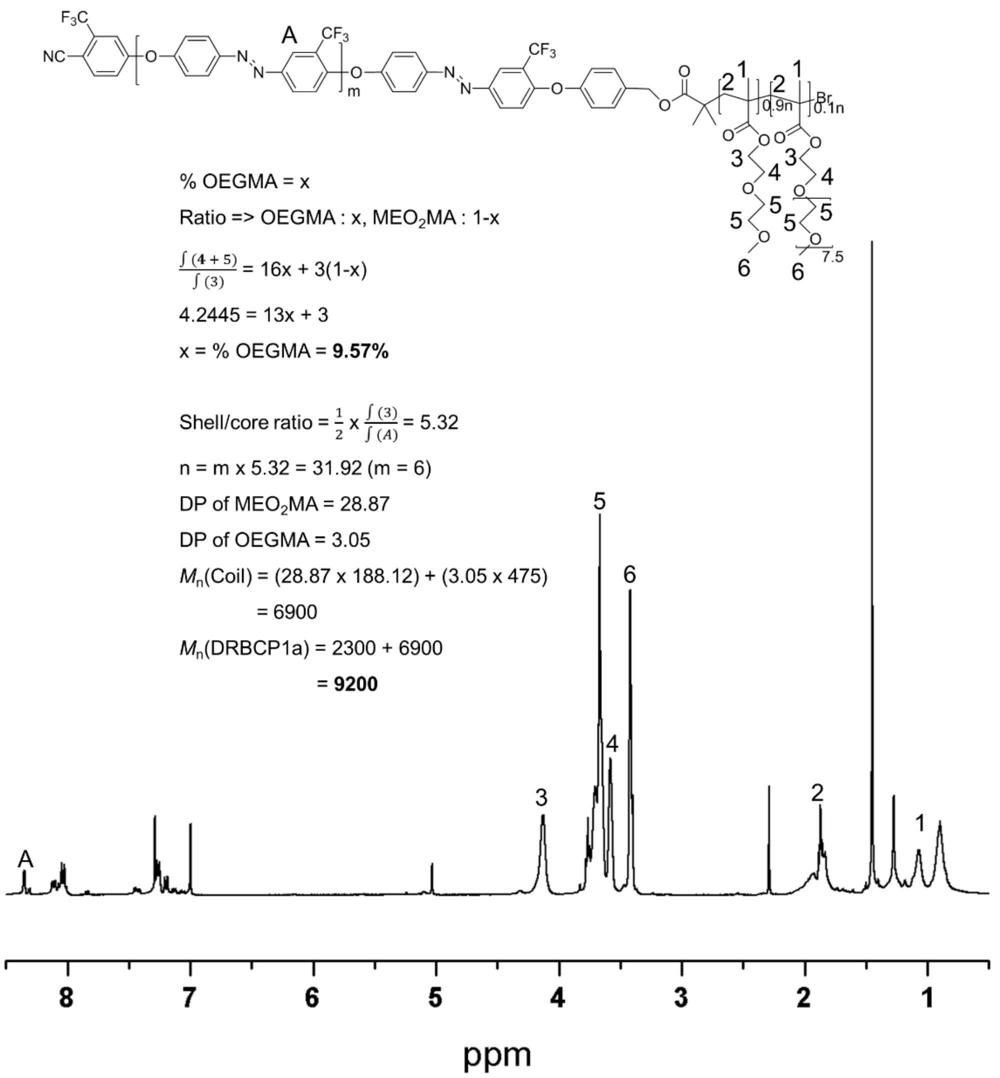

Figure S2. ^{1}H NMR spectrum with calculation of % OEGMA and molecular weight of DRBCP1a in chloroform-*d*.

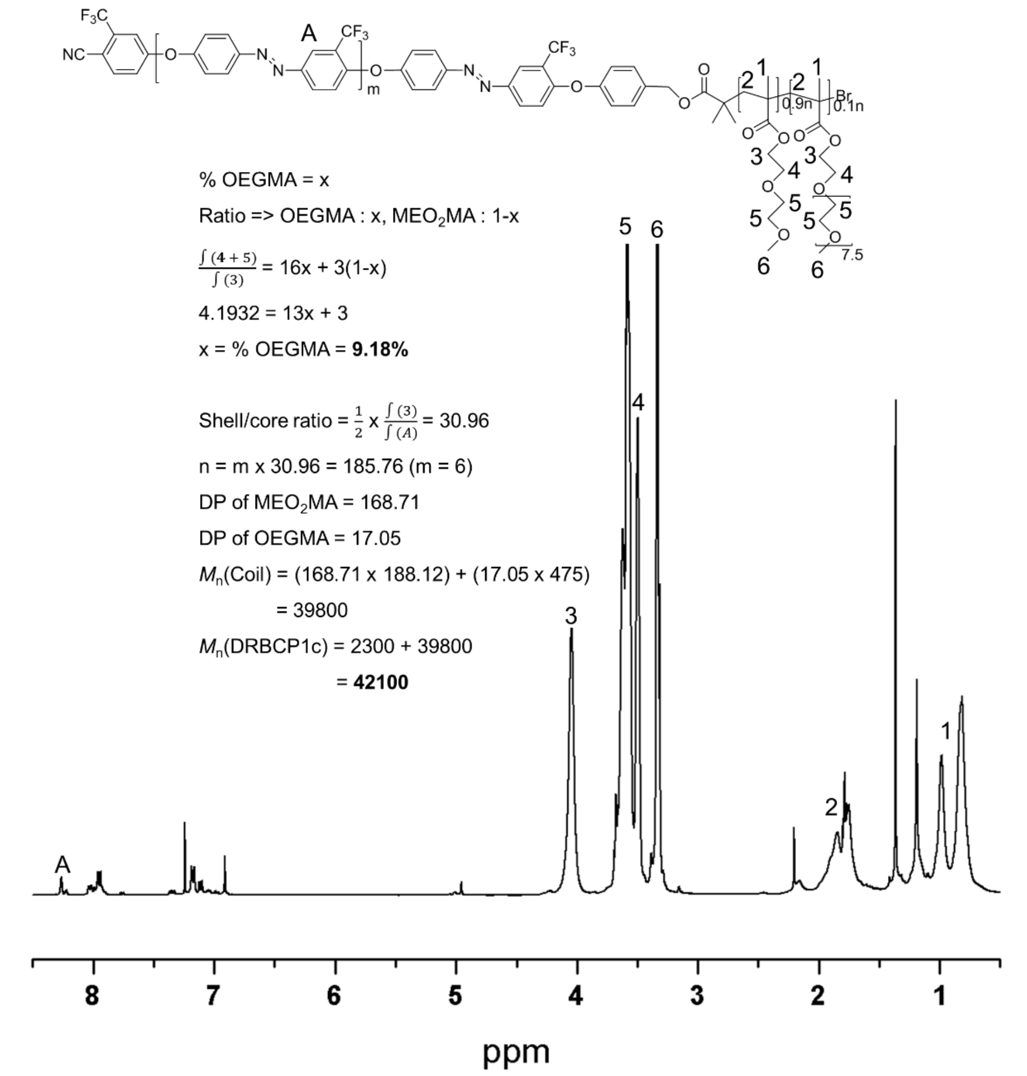

Figure S3. ^{1}H NMR spectrum with calculation of % OEGMA and molecular weight of DRBCP1c in chloroform-*d*.

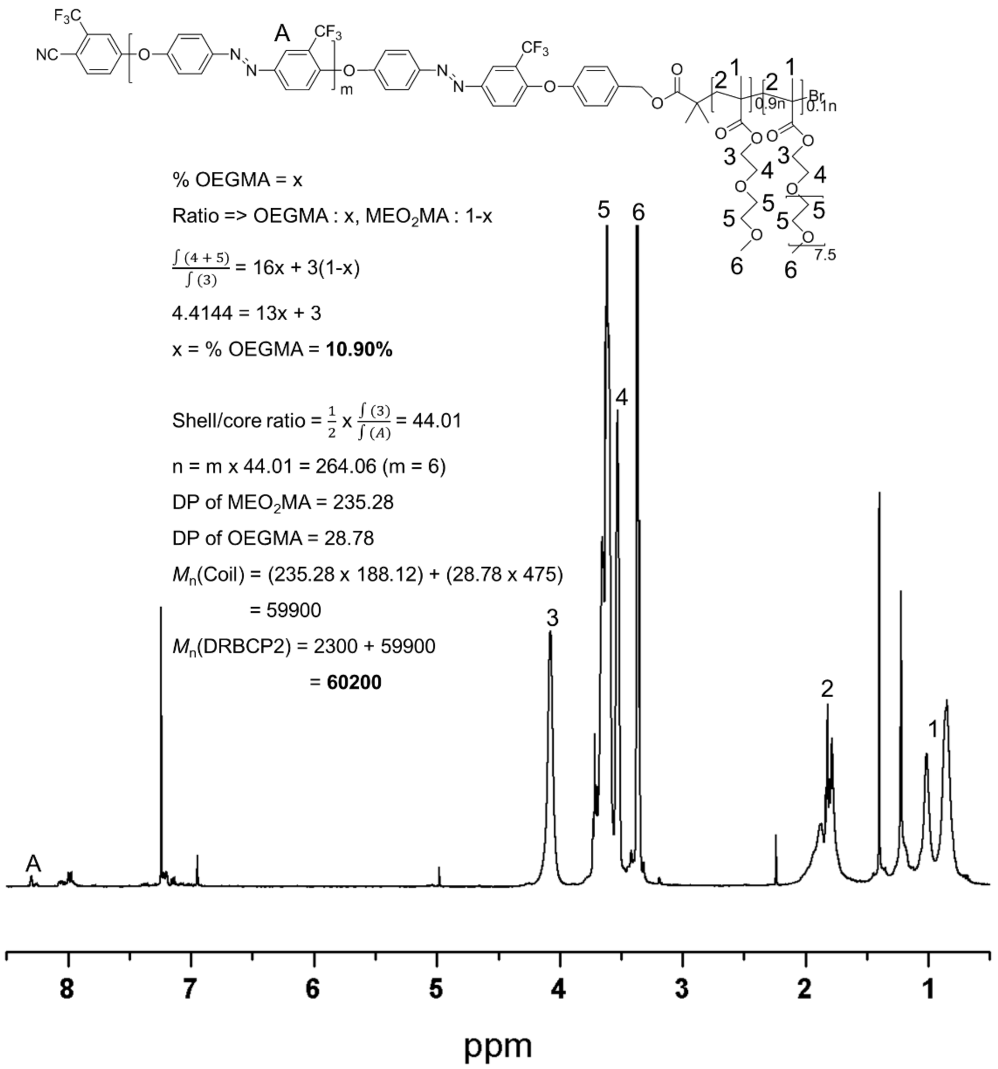

Figure S4. ^{1}H NMR spectrum with calculation of % OEGMA and molecular weight of DRBCP2 in chloroform-*d*.

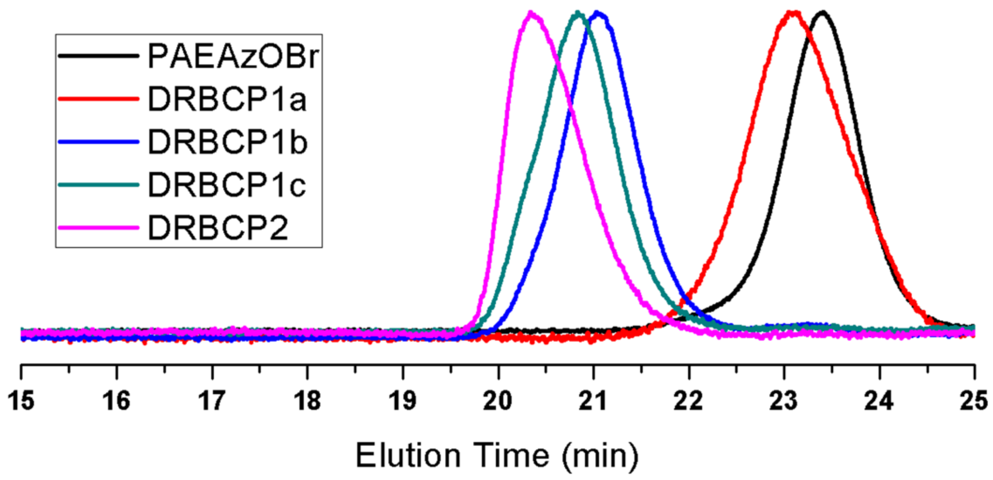

Figure S5. THF-GPC profiles of DRBCPs.

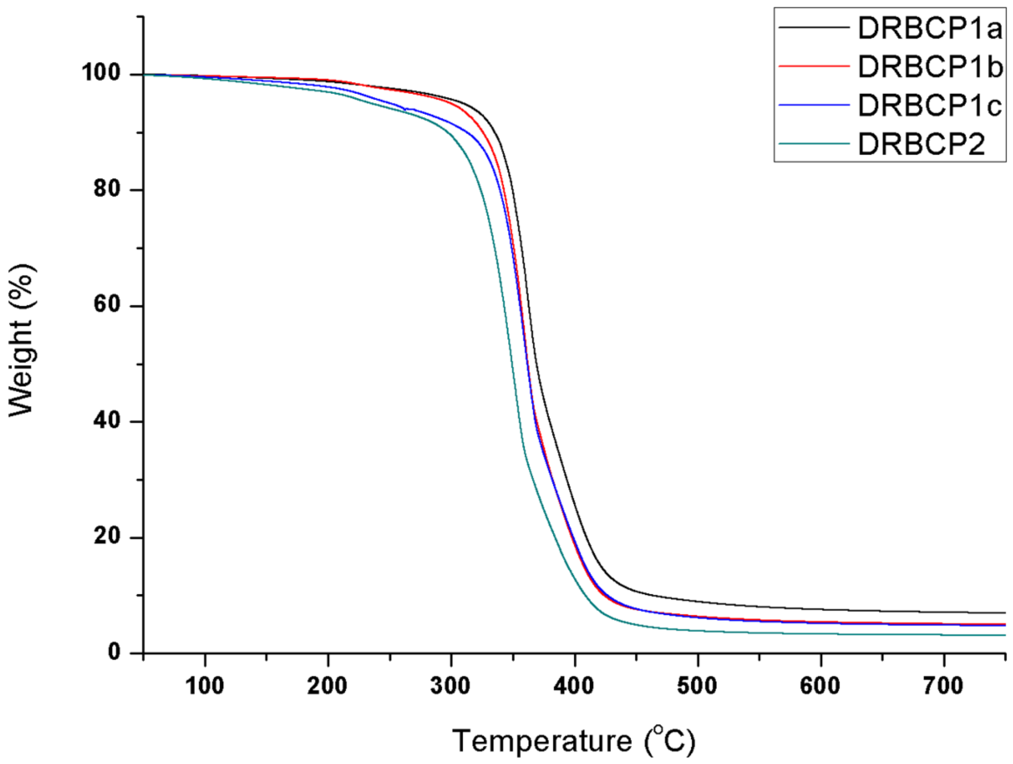

Figure S6. TGA curves of the DRBCPs with a heating rate of 10 °C/min under nitrogen flow.

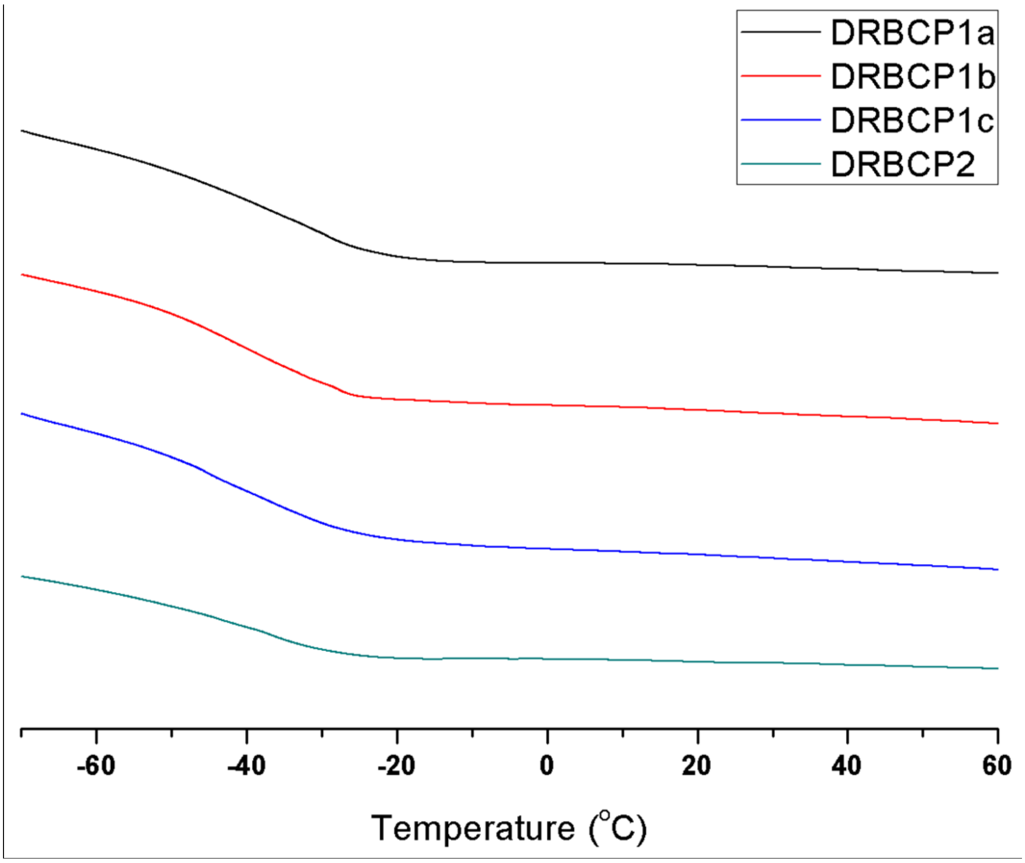

Figure S7. DSC diagrams of the DRBCPs at the second scan with a heating rate of 5 °C/min under nitrogen flow.

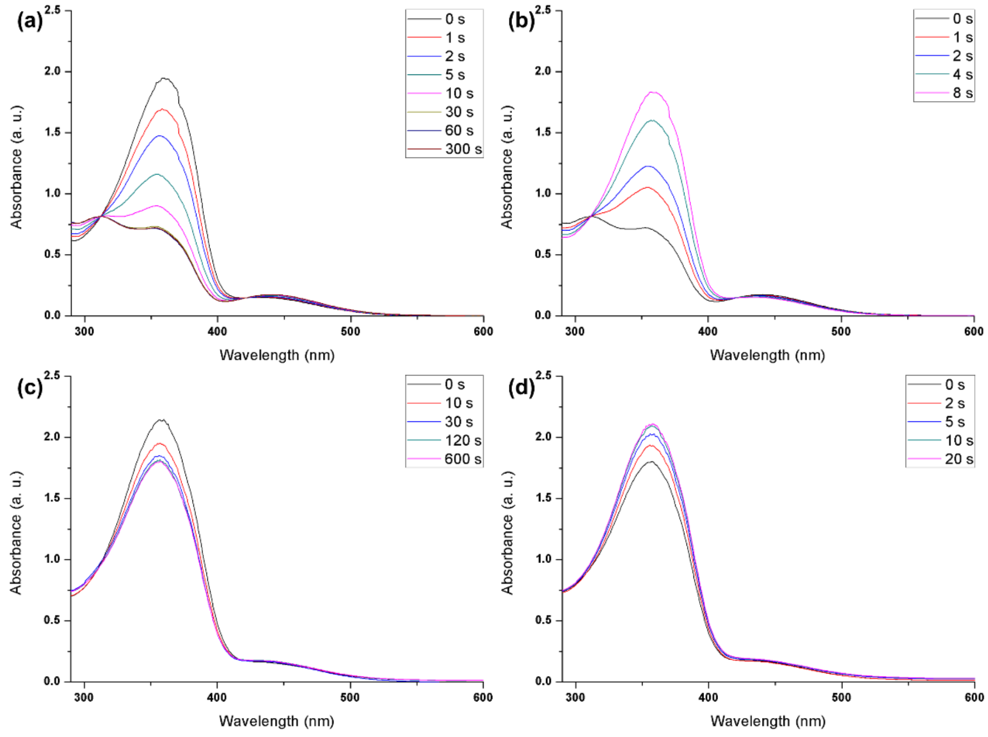

Figure S8. UV-vis absorption spectral changes of DRBCP1c (a, b) in THF solution and (c, d) in aqueous solution. Irradiation of (a, c) UV light and (b, d) visible light subsequently were conducted.

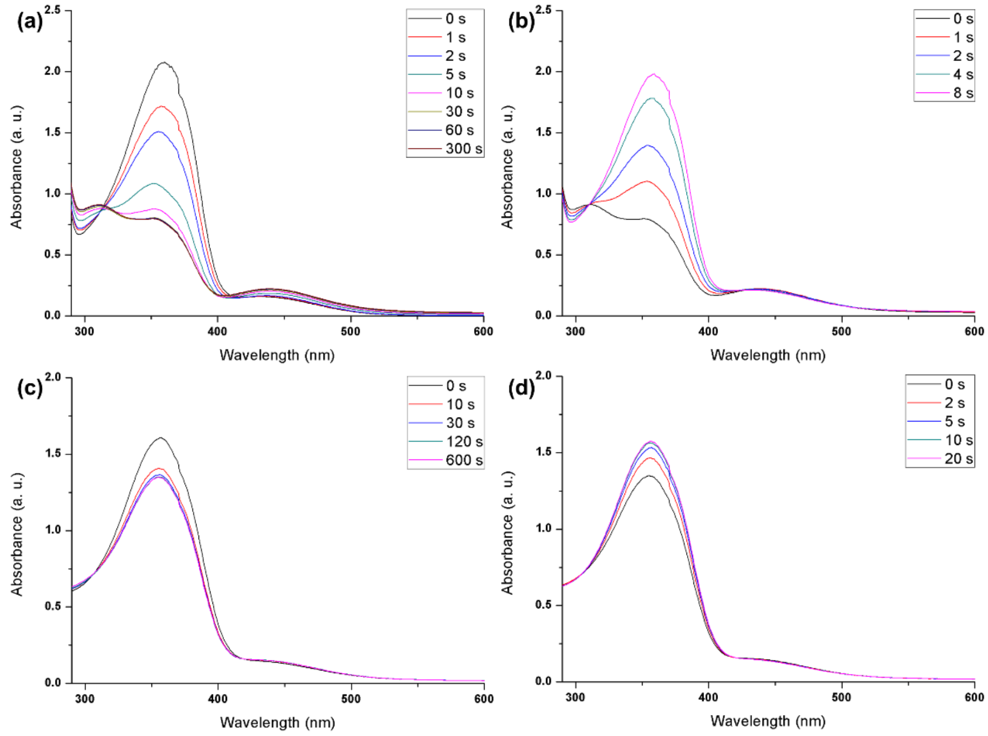

Figure S9. UV-vis absorption spectral changes of DRBCP2 (a, b) in THF solution and (c, d) in aqueous solution. Irradiation of (a, c) UV light and (b, d) visible light subsequently were conducted.

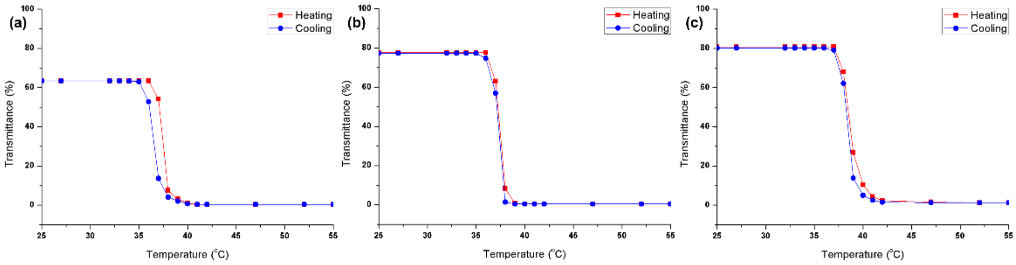

Figure S10. Reversible thermo-responsive behaviors of DRBCPs in aqueous solution (5 mg/mL). (a) DRBCP1b. (b) DRBCP1c. (c) DRBCP2.

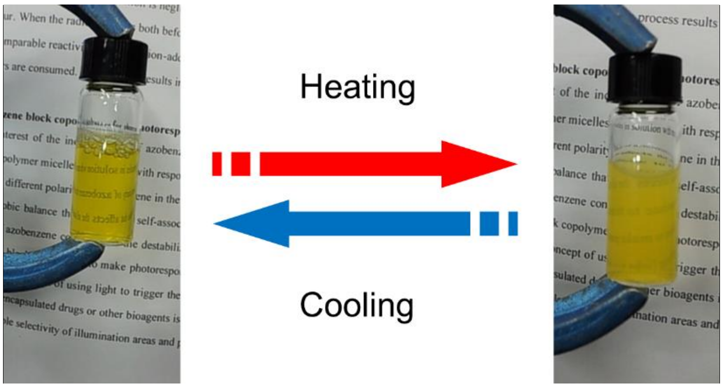

Figure S11. Visualization of transmittance change by controlling temperature.
